# Mining and visualization of microarray and metabolomic data reveal extensive cell wall remodeling during winter hardening in Sitka spruce (*Picea sitchensis*)

**DOI:** 10.3389/fpls.2012.00241

**Published:** 2012-10-29

**Authors:** Ruth Grene, Curtis Klumas, Haktan Suren, Kuan Yang, Eva Collakova, Elijah Myers, Lenwood S. Heath, Jason A. Holliday

**Affiliations:** ^1^Department of Plant Pathology, Physiology, and Weed Science, Virginia TechBlacksburg, VA, USA; ^2^Genetics, Bioinformatics and Computational Biology Program, Virginia TechBlacksburg, VA, USA; ^3^Department of Forest Resources and Environmental Conservation, Virginia TechBlacksburg, VA, USA; ^4^Department of Computer Science, Virginia TechBlacksburg, VA, USA

**Keywords:** microarray, Sitka spruce, carbon metabolism, cell walls, adaptation mechanisms, visualization

## Abstract

Microarray gene expression profiling is a powerful technique to understand complex developmental processes, but making biologically meaningful inferences from such studies has always been challenging. We previously reported a microarray study of the freezing acclimation period in Sitka spruce (*Picea sitchensis*) in which a large number of candidate genes for climatic adaptation were identified. In the current paper, we apply additional systems biology tools to these data to further probe changes in the levels of genes and metabolites and activities of associated pathways that regulate this complex developmental transition. One aspect of this adaptive process that is not well understood is the role of the cell wall. Our data suggest coordinated metabolic and signaling responses leading to cell wall remodeling. Co-expression of genes encoding proteins associated with biosynthesis of structural and non-structural cell wall carbohydrates was observed, which may be regulated by ethylene signaling components. At the same time, numerous genes, whose products are putatively localized to the endomembrane system and involved in both the synthesis and trafficking of cell wall carbohydrates, were up-regulated. Taken together, these results suggest a link between ethylene signaling and biosynthesis, and targeting of cell wall related gene products during the period of winter hardening. Automated Layout Pipeline for Inferred NEtworks (ALPINE), an in-house plugin for the Cytoscape visualization environment that utilizes the existing GeneMANIA and Mosaic plugins, together with the use of visualization tools, provided images of proposed signaling processes that became active over the time course of winter hardening, particularly at later time points in the process. The resulting visualizations have the potential to reveal novel, hypothesis-generating, gene association patterns in the context of targeted subcellular location.

## Introduction

Annual oscillations between growth and dormancy are unique features of woody perennials adapted to life in temperate and boreal regions worldwide (Rohde and Bhalerao, 2007). The timing of these transitions is driven by day length and temperature, and underlies the basic physiological principle that growth and substantial freezing tolerance are not compatible (Weiser, 1970). While cold and freezing tolerance are sometimes confounded in the literature, there are important differences between these two processes. Many annual plants have gene regulatory networks that respond to low, above-freezing temperatures (chilling), but because extra- and intra-cellular ice formation presents a suite of different physiological challenges compared to those presented by chilling, many of these same plants are killed by temperatures much below freezing. While much is known about the physiological basis for cold and freezing injury and tolerance in both annuals and perennials, and substantial effort has been devoted to uncovering the genes involved in transient cold tolerance in annuals (Smallwood and Bowles, 2002), much less is known about the genetic and genomic bases for acclimation to freezing in perennials. The distinction between the two is important for two reasons. First, as mentioned above, freezing is a fundamentally different stress than chilling, and second, plant species that have adopted the perennial habit do not wait for cold temperatures to acclimate. Instead, they use annual environmental cues, most often a critical night length, to begin acclimating to impending freezing temperatures well in advance of the need for freezing tolerance (Weiser, 1970). In this way, both the upstream regulatory network and downstream molecular physiologies of seasonal freezing tolerance are different than the cognate processes for cold tolerance in annuals.

In addition to the different metabolic remodeling involved in chilling and freezing tolerance in annuals and perennials, respectively, a distinction can be made between deciduous and evergreen perennials. Several studies have characterized the transcriptional and metabolic remodeling that occurs in angiosperm trees, mostly in poplar (*Populus*) species, during the transition from growth to dormancy. Ruttink et al. (2007) monitored gene expression and soluble metabolites in hybrid poplar (*Populus tremulax P. alba*) vegetative buds for 6 weeks following the transition to short days and proposed a model for bud dormancy acquisition in which the initial long night signal progresses through ethylene and abscisic acid (ABA)-mediated processes finally leading to endodormancy. As vegetative buds are the structure that perceive and regulate dormancy, the transcriptome and metabolome changes described in that study comprise both processes related to bud dormancy as well as freezing acclimation. Druart et al. (2007) took a similar approach to characterizing molecular events of the growth and dormancy cycle in the cambial meristem of European aspen (*P. tremula*), and identified several distinct stages of transcriptional and metabolic remodeling associated with freezing acclimation (Uemura et al., 2006). Many canonical chilling tolerance-related transcripts were induced well before the onset of freezing temperatures, providing a link to phenotypic measurements in trees showing that deep freezing tolerance is typically acquired before it is needed in most years.

Whereas the primary role of freezing tolerance in angiosperm trees is to protect the cambial meristem and vegetative buds, in evergreen species, which do not shed their leaves in winter, freezing tolerance involves both acclimation of the cambial and shoot meristems, as well as the leaves. Most angiosperm perennials that inhabit cold climates have broad leaves, and few retain them during winter. In contrast to angiosperms, nearly all gymnosperms from cold climates retain their leaves (needles) year-round (with the notable exception of larch species (*Larix spp*.). In addition, gymnosperm trees tend to be ecologically dominant in very cold climates, and understanding the molecular events that allow such species to retain their photosynthetic tissue (although photosynthesis may be greatly reduced) in the face of extreme cold is a basic biological question that cannot be addressed in angiosperms such as *Populus* spp. Several studies have used gene expression microarrays to study the transition to dormancy in gymnosperms in both the *Picea* and *Pinus* genera. In the first such study, Joosen et al. (2006) (Ruonala et al., 2008; Mohamed et al., 2010) employed a 1.5 k cDNA microarray to identify a number of candidate genes for freezing tolerance. More recently, white spruce (*Picea glauca*) stems were sampled at multiple time points following the transition to short days and quantified for both transcript (11 k cDNA microarray) and protein (2D-PAGE) levels (Galindo Gonzalez et al., 2012). Interestingly, several putative spruce orthologs of the Arabidopsis flowering-time pathway were differentially expressed, strengthening the link between this pathway and light-mediated dormancy signaling in perennials (Bohlenius et al., 2006). To date, the only study of transcriptome remodeling in photosynthetic tissue during freezing tolerance acquisition in a gymnosperm was in Sitka spruce (*Picea sitchensis)*, for which five time points from late summer to early winter were sampled and gene expression monitored using a 21.8 k array (Holliday et al., 2008). Holliday et al. focused primarily on downstream cryoprotective genes, lipids, soluble carbohydrates, and upstream signaling components (e.g., calcium, daylength perception) related to freezing acclimation. However, they did not discuss in detail genes assigned to several “gene ontology (GO)” terms that were overrepresented, including “transport,” “transporter activity,” “endoplasmic reticulum,” and “golgi apparatus.” As little is known about the role of the endomembrane system with regard to the process of freezing acclimation, we sought to more deeply probe expression patterns and possible function of the relevant genes. In the current paper, we employ several novel analytical tools to dissect the role of the secretory system, and its relationship to cell wall remodeling, with respect to cold hardiness in spruce.

## Methods

### Plant material, microarray analysis, and metabolite profiling

The plant material and resulting gene expression data for this study are described in Holliday et al. (2008). Briefly, a common garden comprising Sitka spruce originating from 17 geographic populations was grown in an outdoor, raised-bed common garden at Vancouver, BC, Canada. In the fourth season of growth, plants from a population originating near the center of the species range were sampled for RNA extraction and phenotyped by an artificial freeze test that uses electrolyte leakage as a proxy for cell death in sectioned needles frozen at several test temperatures. In this case, the index of injury (I_t_) at −10°C was used. Needle tissue was sampled at five time points: August 30, October 18, November 22, December 1, and December 13 (2004). Holliday et al. (2008) showed that using this freeze test, the plants began acclimating to freezing after October 18, and reached maximal levels of hardiness on the fourth time point (December 1). Hereafter, expression changes associated with these time points are referred to as TP1 for the ratio of October 18: August 30, TP2 for the ratio of November 22: August 30, and so on. RNA was extracted from these needles, followed by cDNA synthesis and hybridization to a 21.8 k spruce cDNA microarray. Slides were scanned and spot intensity quantified using ImaGene software (BioDiscovery, Inc., El Segundo, CA). Data were background-subtracted, normalized by variance stabilizing normalization (VSN) (Huber et al., 2002), and significant changes in gene expression were identified using a linear mixed-effects model as described in Holliday et al. (2008). *P*-values for each gene-by-treatment effect were adjusted for the false discovery rate (FDR) (Storey and Tibshirani, 2003). In addition to transcriptome analysis, data on soluble metabolites quantified by GC-MS analysis were taken from Dauwe et al. for the same samples (Dauwe et al., 2012). All statistical analyses were carried out using the R statistical package (www.r-project.org).

### Use of MapMan to reveal pathways enriched over the hardening period

The MapMan tool facilitates the classification and statistical analysis of transcripts and metabolites into hierarchical categories (known as bins) in a manner that avoids the redundancy present in other commonly used ontologies (Usadel et al., 2009); therefore, the tool provides an additional level of analysis beyond the functional enrichment of typical GO categories. A further, in–house modification of MapMan was made to allow visual comparisons of changes over time by the inclusion of the ability to view multiple instances of a bin simultaneously. Using this viewpoint, the user is presented with multiple copies of the same bin, where each copy displays the expression of transcripts or metabolites annotated to the bin for the selected condition. This visualization technique, which is similar to Tufte's small multiples concept (Tufte, 1983), is based on the principle that different visualization techniques may be more or less effective depending on the data analysis task that is being performed (Saraiya et al., 2005). As an additional analytical viewpoint, the user is presented with a list of each gene or metabolite annotated to the visualized bin that demonstrates the change in each entity individually.

### Functional gene ontology analysis

Functional properties of the differentially expressed clones were categorized based on the GO database (Ashburner et al., 2000) using ontologizer (Bauer et al., 2008). First, putative orthologs of Sitka spruce genes on the microarray were identified using a custom BLAST (Altschul et al., 1997) database comprised of all the *Arabidopsis* sequences annotated by TAIR (Lamesch et al., 2012) and BLAST executables (http://www.ncbi.nlm.nih.gov) were integrated to automate this process. Over-represented GO terms were identified using the parent-child-union algorithm implemented in Ontologizer. This method is preferred over the other methods because it scores each GO term considering the presence of the parent term rather than taking only the individual GO contributions into account. The Westfall-Young single step error correction was used to adjust for multiple testing in the identification of significant GO terms (Westfall and Young, 1993). An FDR of less than 1% was considered statistically significant.

### Automated gene association network generation using ALPINE

As genes that showed large and statistically significant changes in expression across time points were identified, the Automated Layout Pipeline for Inferred NEtworks (ALPINE) tool was used to provide context regarding the associations among the identified genes and other genes showing statistically significant changes in expression in the data set. The ALPINE tool, implemented as a plugin for the Cytoscape visualization environment (Shannon et al., 2003), performs the following steps when processing a set of query genes: first, the genes are used as a query set to generate a network of gene associations (co-expression, co-localization, genetic interaction, physical interaction, and more) using the GeneMANIA (Montojo et al., 2010) Cytoscape plugin; second, the resulting gene association network is then filtered to include only those genes that showed a statistically significant expression value in a user-defined minimum number of time points; finally, the filtered network is organized into a layout that uses node color to represent GO molecular function annotations, with nodes drawn over a background image of cellular components using GO cellular component annotations. The annotations and layout are executed using the Mosaic Cytoscape plugin (Zhang et al., 2012). (Specific use of the ALPINE tool is further described in the section “Results”).

## Results

### GO enrichment reveals a relationship between endomembrane trafficking and cell wall remodeling during freezing acclimation

**Figure 1:**
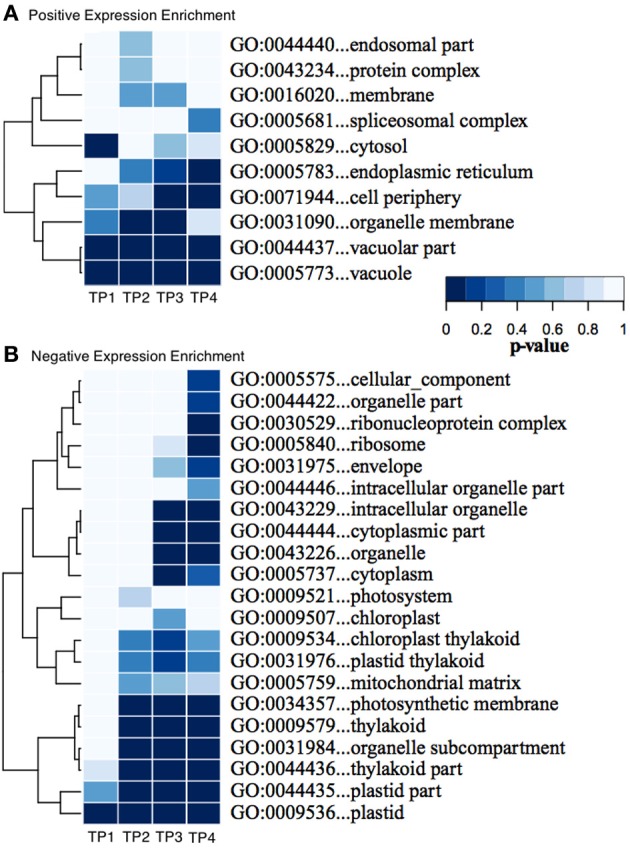
**(A,B)** Enrichment of GO “Cellular Component” categories across the time series of winter hardening. The GO tool Ontologizer was used to assess statistical enrichment of genes related to the cellular component ontology. Only GO terms with significant enrichment for at least one time point are shown. Colors correspond to probability values for individual terms.

**Figure 2:**
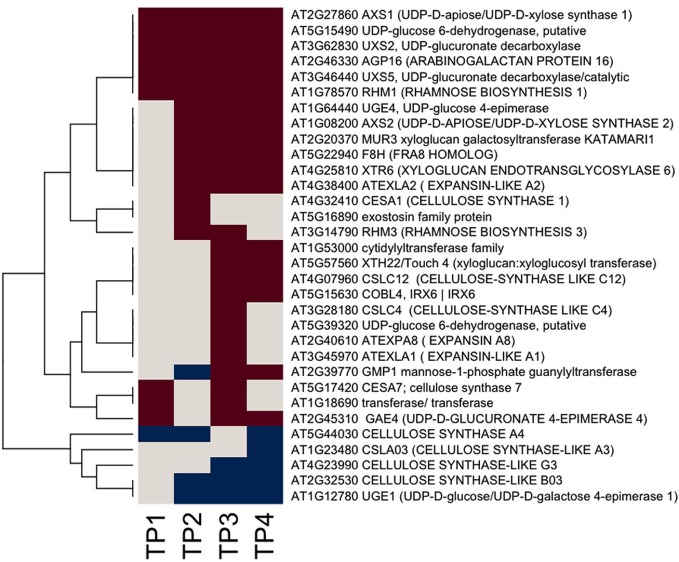
**Heat map of significantly responsive cell wall associated genes across the time course of winter hardening.** The gene expression levels across the rows are hierarchically clustered. Magenta cells represent significant positive expression changes, blue cells represent significant negative changes, and off-white cells represents no change or no statistically significant change.

We used Ontologizer software to perform GO enrichment analysis across cell components (Figures 1A,B), which revealed that genes associated with endomembrane function were up regulated during the time course of winter hardening (Figure 1A). Since most cell wall components are synthesized in and transported by the endomembrane secretory system (Carpita, 2011), results for the two broad categories were examined together. In Figure 2, the MapMan bin for each gene and an indication of when a statistically significant change in expression occurred are displayed in heat map form. A distinction between genes annotated as “cellulose synthase” (CES) vs. “cellulose synthase-like” (CSL) enzymes should be made, as CES are involved in the synthesis of cellulose, while CSL are involved in the synthesis of mannan oligomers and xyloglucans (Davis et al., 2010). CESA1 (At4g32410) showed no changes in transcript levels, except up-regulation in TP2 while CESA4 (At5g44030) was down-regulated in TP1, 2, and 4. In contrast, the xylem-specific CES IRX3 (CESA7, At5g17420) was up-regulated in TP1 and 3. Overall, CSL genes showed no changes in expression in TP1 and 2, but CSL12 was up-regulated and CSLG4 down-regulated in TP3 and 4. Both CSLB03 and E1 were consistently down-regulated through TP2 and 4. Cobra-like O-glycosyl hydrolase 4 (At5g15630), involved in xylan and secondary cell wall biosynthesis (Oikawa et al., 2010), exhibited an elevated expression between TP3 and 4. Table 1 summarizes changes in the levels of metabolites relevant to cell wall biosynthesis during winter hardening. Levels of the metabolite glucose-6-phosphate, a precursor of the cellulose biosynthetic pathway, showed an increase over the time course, while glucose-1,6-bisphosphate exhibited slightly higher but not significant levels in TP1 through TP3, followed by a decline at TP4. The levels of arabinose released during cell wall degradation decreased at TP1 and remained low for the remainder of the time course.

**Table 1:** **Changes in steady-state metabolite levels during winter hardening**.

**Metabolite**	**TP1**	**TP2**	**TP3**	**TP4**
Glucose-6-phosphate	1.25	**1.89**	**2.23**	**2.07**
Glucose-1,6-phosphate	1.02	1.06	1.19	−1.36
Arabinose	**−2.69**	**−2.24**	**−2.45**	−1.92

Significant changes (P < 0.05 for a given contrast; Dauwe et al., 2012) are shown in bold.

A somewhat different pattern was observed in the case of genes in the subcategories corresponding to the biosynthesis of cell wall carbohydrate precursors (Figure 2), where an overall up-regulation and fewer decreases in gene expression over the time course were observed in the case of this group. UDP-D-glucose/UDP-D-galactose 4-epimerase 1 (UGE1, At1g12780) converts UDP-galactose to UDP-glucose, and it was the only gene exhibiting a consistent down-regulation from TP2 toTP4. In contrast, UGE4 (At1g64440) showed an opposite trend to UGE1. Most genes encoding different enzymes involved in cellulose biosynthesis showed increases at TP1, with a consistent pattern of increases in transcript levels thereafter over the remainder of the time course. This group included one of the genes encoding UDP-glucose-6-dehydrogenase (At5g15490) that catalyzes the first step in the cellulose biosynthesis pathway, specifically the oxidation of UDP-glucose to UDP-glucuronate, and a number of genes encoding enzymes of the subsequent steps. These enzymes included two UDP-glucuronate decarboxylases (At3g62830 and At3g46440) and UDP-glucuronate 4-epimerase 4 (At2g45310), which was consistently up-regulated except for TP2. Similar trends were observed for genes encoding enzymes that participate in the synthesis of the subsequent carbohydrate precursors of the cell wall from UDP-glucuronate, including UDP-mannose, -rhamnose, -xylose, and -apiose. Genes encoding GDP-mannose 4,6-dehydratase 1 (At5g66280), rhamnose biosynthesis 1 (At1g78570), and UDP-apiose/UDP-xylose synthase 1 (At2g27860) remained increased at and beyond TP1. Other homologous genes encoding these enzymes were up-regulated less consistently during different time points.

Xyloglucans are major components in hemicelluloses in primary cell walls and are synthesized via various glycosyltransferases. The gene encoding xyloglucan:xyloglucosyltransferase (At5g57560, XTH22) that catalyzes the modification of xyloglucan oligomers was up-regulated during TP3 and 4, while xyloglucanendotransglycosylase 6 (XTR6, At4g25810) and xyloglucangalactosyltransferase MUR3 [At2g20370, Golgi (Chevalier et al., 2010)] were also up-regulated at TP2. F8H glycosyltransferase (At5g22940) is involved in the biosynthesis of glucuronoxylans in Arabidopsis that are part of the secondary cell walls in the xylem (Lee et al., 2009). The corresponding gene in Sitka spruce showed up-regulation patterns comparable to XTR6 and MUR3. Some members of the expansin gene family showed up-regulation, e.g., expansin A8 and expansin-like A1 in TP3, while expansin-like A2 from TP2 to 4. The arabinogalactan protein (AGP)-encoding gene family showed strikingly diverse patterns of expression over the time course of winter hardening, with an AGP16 homolog increasing during all time points in winter hardening.

### Secretory system

**Figure 3:**
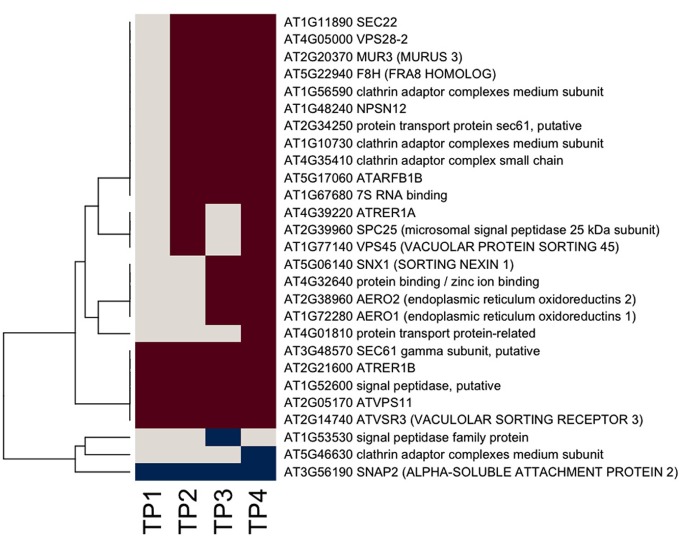
**Heat map of significantly responsive endomembrane/secretory pathway associated genes across the time course of winter hardening.** The gene expression levels across the rows are hierarchically clustered. Magenta cells represent significant positive expression changes, blue cells represent significant negative changes, and off-white cells represent no change or no statistically significant change.

Given that the biosynthesis of cell wall matrix polysaccharides and other cell wall components takes place in the Golgi apparatus (Driouich et al., 2012), an examination of the responsiveness of genes encoding proteins associated with the endomembrane system and associated secretory processes was warranted. The assignment of responsive genes to the “protein targeting secretory pathway” bin in MapMan was taken as a starting point, and the assignment of those genes were further refined using information from PubMed on individual genes, together with information from GO. Results are shown in heat map form in Figure 3. The majority of responsive genes associated with the secretory system were up-regulated during the time course of hardening, suggesting extensive secretory activity. One group of genes was up regulated across the entire time course. This included At3g48570, encoding the gamma subunit of SEC61, a protein transporter, (At2g14740), a vacuolar sorting receptor, and MUR3 (At2g20370). Another group showed up-regulation from TP2 onwards, including At1g11890, encoding SEC22 (El-Kasmi et al., 2011), whose function has been shown to be essential for the integrity of the Golgi (Table 3).

**Figure 4:**
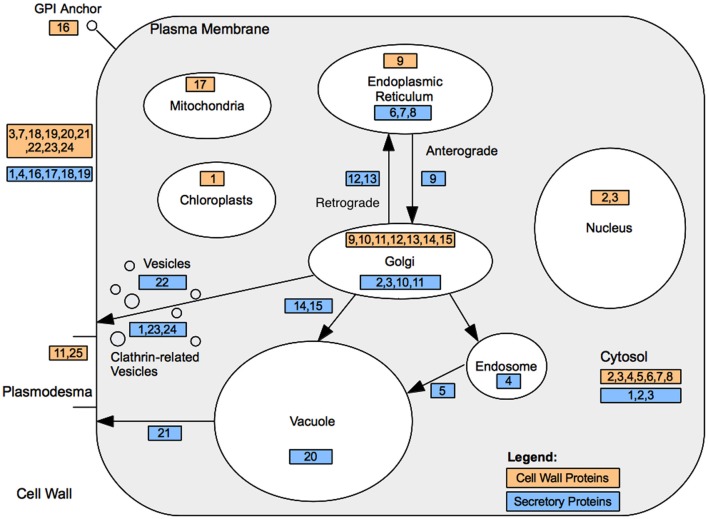
**Cell wall and secretory protein localization.** Cell wall associated protein locations are denoted by their ID within light orange shaded rectangles. Endomembrane/secretory pathway associated proteins are represented by their ID within light blue rectangles. Further details are presented in Tables 2 and 3.

Genes encoding secretory pathway proteins and cell wall associated functions that were significantly up-regulated at a minimum of one time point are grouped according to compartment, and direction of movement of the protein is indicated, where known (Figure 4; Tables 1 and 2). It is apparent that several genes encoding proteins targeted to the cell membrane and another set destined for the Golgi apparatus responded positively during winter hardening, with comparatively little response among peroxisomal or nuclear-targeted gene products. In the case of the cell membrane, five responsive genes associated with secretion were up-regulated. The other four up-regulated genes encoding homologs of transport/secretion proteins showed an initial change, reflected in the TP2/TP1 comparison, with increases shown over the remaining time points. In the case of proteins localized to the Golgi, ten genes responded positively over the time course. The positive responders included ATRER1A and ATRER1B (At2g21600 and At4g39220, respectively), which facilitate retrograde transport from the Golgi to the ER. Three genes, encoding proteins associated with anterograde transport, from ER to Golgi, were up-regulated, including ATSEC22 (At1g11890). In the case of the other two genes in this category, At4g01810 and At4g32640, an initial decrease was observed at the TP2/TP1 comparison, followed by increases between TP3 and 2. Two genes encoding proteins that are targeted to the trans-golgi network (TGN) were also up-regulated over the time course, including one (At1g77140) that is targeted to the tonoplast. Another gene, At2g14740, is annotated as being part of the “Golgi transport complex,” part of the cell membrane, and also targeted to the vacuole. Such complex annotations may reflect the mobility of proteins within the endomembrane system. In the case of the ER, two genes encoding proteins that catalyze thiol-disulfide exchange were also up-regulated.

**Table 2:** **Cell wall associated protein localization**.

**ID**	MapMan Bin (cell wall.^*^)	**Gene ID**	**Description**
**CHLOROPLAST**			
1	Hemicellulose synthesis	AT5G16890	Exostosin family protein
**CYTOSOL**			
2	Precursorsynthesis.UGD	AT5G39320	UDP-glucose 6-dehydrogenase, putative
3	Precursorsynthesis.UGD	AT5G15490	UDP-glucose 6-dehydrogenase, putative
4	Precursorsynthesis.AXS	AT1G08200	AXS2 (UDP-D-APIOSE/UDP-D-XYLOSE SYNTHASE 2)
5	Precursorsynthesis.RHM	AT3G14790	RHM3 (RHAMNOSE BIOSYNTHESIS 3)
6	Precursorsynthesis.AXS	AT2G27860	AXS1 (UDP-D-apiose/UDP-D-xylose synthase 1)
7	Cellwall.precursorsynthesis.UGE	AT1G12780	UGE1 (UDP-D-glucose/UDP-D-galactose 4-epimerase 1)
8	Precursorsynthesis.NDP sugar pyrophosphorylase.GDP mannose	AT2G39770	GMP1 mannose-1-phosphate guanylyltransferase
**ER**			
9	Cellulosesynthesis.cellulose synthase	AT1G23480	CSLA03 (CELLULOSE SYNTHASE-LIKE A3)
**GOLGI**			
10	Precursorsynthesis.UXS	AT3G62830	UXS2, UDP-glucuronate decarboxylase
11	Precursorsynthesis.UGE	AT1G64440	UGE4, UDP-glucose 4-epimerase
12	Hemicellulosesynthesis.xyloglucan.XXXGgalactoseTransferase	AT2G20370	MUR3
13	Hemicellulosesynthesis.glucuronoxylan	AT5G22940	F8H (FRA8 HOMOLOG
9	Cellwall.cellulosesynthesis.cellulose synthase	AT1G23480	CSLA03 (CELLULOSE SYNTHASE-LIKE A3)
14	Cellwall.modification	AT5G57560	XTH22/Touch 4 (xyloglucan:xyloglucosyltransferase)
15	Cellwall.modification	AT4G25810	XTR6 (XYLOGLUCAN ENDOTRANSGLYCOSYLASE 6)
**GPI ANCHOR**			
16	Cell wall proteins.AGPs	AT2G46330	AGP16 (ARABINOGALACTAN PROTEIN 16)
**MITOCHONDRIA**			
17	Precursorsynthesis.KDOpathway.CMP-KDO synthetase	AT1G53000	Cytidylyltransferase family
**NUCLEUS**			
2	Precursorsynthesis.UGD	AT5G39320	UDP-glucose 6-dehydrogenase, putative
3	Precursorsynthesis.UGD	AT5G15490	UDP-glucose 6-dehydrogenase, putative
**PLASMA MEM.**			
7	Precursorsynthesis.UGE	AT1G12780	UGE1 (UDP-D-glucose/UDP-D-galactose 4-epimerase 1)
18	Hemicellulose synthesis	AT1G18690	Transferase/transferase, transferring glycosyl groups
19	Cellulose synthesis	AT3G28180	CSLC4 (CELLULOSE-SYNTHASE LIKE C4)
20	Cellulosesynthesis.cellulose synthase	AT4G32410	CESA1 (CELLULOSE SYNTHASE 1)
21	Cellulosesynthesis.cellulose synthase	AT5G17420	CESA7; cellulose synthase
22	Cellwall.modification	AT2G40610	ATEXPA8 (EXPANSIN A8)
23	Cellwall.modification	AT3G45970	ATEXLA1 (EXPANSIN-LIKE A1)
24	Cellwall.modification	AT4G38400	ATEXLA2 (EXPANSIN-LIKE A2)
14	Cellwall.modification	AT5G57560	XTH22/Touch 4 (xyloglucan:xyloglucosyltransferase)
15	Cellwall.modification	AT4G25810	XTR6 (XYLOGLUCAN ENDOTRANSGLYCOSYLASE 6)
3	Precursorsynthesis.UGD	AT5G15490	UDP-glucose 6-dehydrogenase, putative
**PLASMODESMA**			
11	Precursorsynthesis.UGE	AT1G64440	UGE4, UDP-glucose 4-epimerase
25	Cellulose synthesis	AT4G07960	CSLC12 (CELLULOSE-SYNTHASE LIKE C12)

* This table shows cell wall associated genes exhibiting significant up-regulation at one or more time points organized by cellular location. MapMan bin names are shortened by removing the common prefix “cell wall.”

**Table 3:** **Secretory protein localization**.

**ID**	MapMan Bin (protein.targeting.^*^)	**Gene ID**	**Description**
**CYTOSOL**			
1	Secretorypathway.unspecified	AT5G46630	Clathrin adaptor complexes medium subunit
2	Secretorypathway.golgi	AT4G01810	Protein transport protein-related
3	Secretorypathway.golgi	AT4G32640	Protein binding/zinc ion binding
**ENDOSOME**			
4	Secretorypathway.vacuole	AT5G06140	SNX1 (SORTING NEXIN 1)
**ENDOSOME TO VACUOLE**			
5	Secretorypathway.unspecified	AT4G05000	VPS28-2
**ER**			
6	Secretory pathway.ER	AT2G38960	AERO2 (endoplasmic reticulum oxidoreductins 2)
7	Secretory pathway.ER	AT2G39960	Microsomal signal peptidase 25 kDa subunit
8	Secretory pathway.ER	AT1G72280	AERO1 (endoplasmic reticulum oxidoreductins 1)
**ER TO GOLGI**			
9	Secretorypathway.unspecified	AT1G11890	SEC22
**GOLGI**			
2	Secretorypathway.golgi	AT4G01810	Protein transport protein-related
3	Secretorypathway.golgi	AT4G32640	Protein binding/zinc ion binding
10	Cellwall.hemicellulosesynthesis.xyloglucan.XXXGgalactoseTransferase	AT2G20370	MUR3 (MURUS 3)
11	Cellwall.hemicellulosesynthesis.glucuronoxylan	AT5G22940	F8H (FRA8 HOMOLOG)
**GOLGI TO ER**			
12	Secretory pathway.ER	AT2G21600	ATRER1B
13	Secretory pathway.ER	AT4G39220	ATRER1A
**GOLGI TO VACUOLE**			
14	Secretorypathway.unspecified	AT1G56590	Clathrin adaptor complexes medium subunit
15	Secretorypathway.vacuole	AT1G77140	VPS45 (VACUOLAR PROTEIN SORTING 45)
**PLASMA MEM.**			
16	Secretorypathway.unspecified	AT3G48570	SEC61 gamma subunit, putative
17	Secretorypathway.unspecified	AT1G48240	NPSN12
18	Secretorypathway.unspecified	AT1G52600	Signal peptidase, putative
1	Secretorypathway.unspecified	AT5G46630	Clathrin adaptor complexes medium subunit
19	Secretorypathway.unspecified	AT2G34250	Protein transport protein sec61, putative
4	Secretorypathway.vacuole	AT5G06140	SNX1 (SORTING NEXIN 1)
**VACUOLE**			
20	Secretorypathway.vacuole	AT2G05170	ATVPS11
**VACUOLE TO PLASMA MEM.**			
21	Secretorypathway.unspecified	AT3G56190	SNAP2 (ALPHA-SOLUBLE ATTACHMENT PROTEIN 2)
**VESICLES (GOLGI TO PLASMA MEM.)**			
22	Secretorypathway.vacuole	AT2G14740	ATVSR3 (VACULOLAR SORTING RECEPTOR 3)
**VESICLES (GOLGI TO PLASMA MEM.) -CLATHRIN**			
1	Secretorypathway.unspecified	AT5G46630	Clathrin adaptor complexes medium subunit
23	Secretorypathway.unspecified	AT1G10730	Clathrin adaptor complexes medium subunit
24	Secretorypathway.unspecified	AT4G35410	Clathrin adaptor complex small chain
**UNSPECIFIED**			
25	Secretorypathway.unspecified	AT1G53530	Signal peptidase I family protein
26	Secretorypathway.unspecified	AT5G17060	ATARFB1B
27	Secretorypathway.unspecified	AT1G67680	7S RNA binding

* This table shows protein-targeting associated genes exhibiting significant up-regulation at one or more time points organized by cellular location. MapMan bin names are shortened by removing the common prefix “protein.targeting.”

### Hormone-related responses

**Figure 5:**
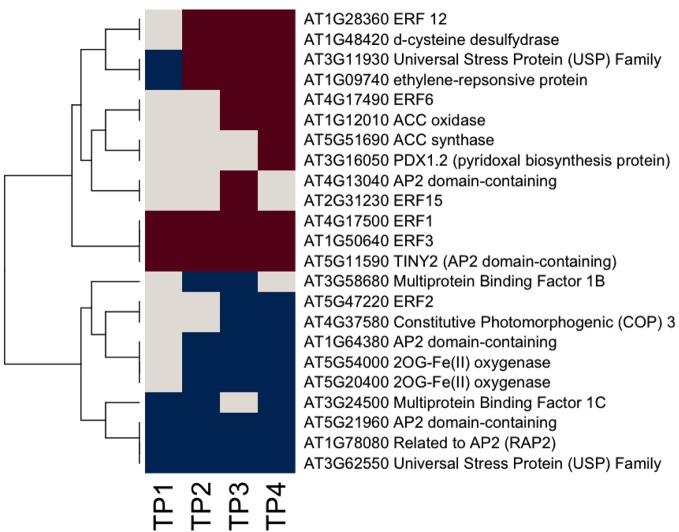
**Ethylene-related gene responses across the time course of winter hardening.** The gene expression levels across the rows are hierarchically clustered. Magenta cells represent significant positive expression changes, blue cells represent significant negative changes, and off-white cells represent no change or no statistically significant change.

**Figure 6:**
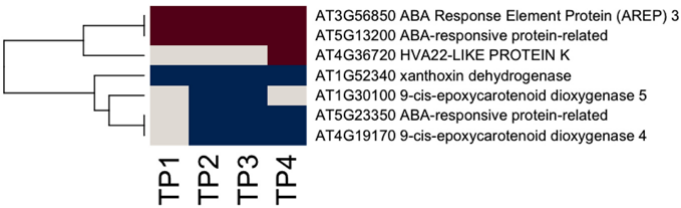
**ABA-related gene expression across the time course of winter hardening.** The gene expression levels across the rows are hierarchically clustered. Magenta cells represent significant positive expression changes, blue cells represent significant negative changes, and off-white cells represent no change or no statistically significant change.

Ethylene synthesis and signaling appear to be active throughout the time course (Figure 5). In contrast, fewer ABA-related genes responded over the time course, including two members of the nine *cis*-epoxycarotenoid dioxygenase (NCED4 and NCED5), which were down regulated, although there is a suggestion that ABA-mediated signaling may have become active (Figure 6). MapMan analysis reflected this, with more responsive genes in the case of the ethylene bin in MapMan than there were for ABA. This is reflected in the up-regulation of five ethylene response factor (ERF) genes (*ERF1, 3, 6, 12*, and *15*) and suggests an increase in ethylene levels up to TP3, followed by maintenance through TP4. In contrast, two genes encoding members of the 2OG-Fe(II) oxygenase gene family, enzymes that degrade ethylene, were down-regulated. Three other ethylene-associated genes that were down-regulated include *HOOKLESS1*, and two members of the multi-protein bridging factor (MBF) gene family, *MBF1B* and *C*.

### Genes associated with highly responsive ethylene signaling, and cell wall related genes later in winter hardening

**Figure 7:**
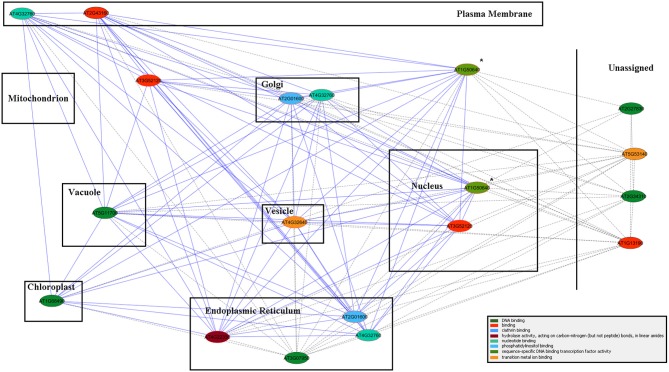
**ALPINE gene association network results for ERF3 in a cellular component context.** Edges represent “guilt by association” between the two connected genes, and node color represents GO molecular function annotation (the color legend is located in the bottom right corner). Due to a lack of specificity in the version of GO cellular component annotations provided by Mosaic, cellular component assignments, as provided directly by GO, were drawn in place of the default Mosaic background image. The query gene is indicated by the “^*^” symbol, and genes with multiple cellular component annotations appear multiple times in the network.

Table 4 lists 13 genes, with their respective MapMan bins, that showed the largest up-regulation over the time course, especially at the later time points. This was true of the gene encoding GPI-anchored AGP16, which may have a role in signaling across the plasma membrane, the transcription factor ERF3 (*ETHYLENE RESPONSIVE ELEMENT BINDING FACTOR 3*), and *TCH4*. The ALPINE pipeline was used to visualize documented “guilt by association” relationships for *ERF3*, reasoning that this strategy might reveal clues concerning major, later, ethylene-related events in winter hardening that are not apparent using our other strategies. Using *ERF3* as the input gene, mining gene expression data obtained for TP3 and 4 resulted in the identification of 11 positively expressed genes related to the input gene through “guilt by association.” Figure 7 shows the result, which is based on co-expression, protein–protein interactions, and genetic interactions in the GeneMANIA database only. Table 5 provides a more detailed notes on the genes highlighted in Figure 7. Several genes encoding proteins located in the endomembrane system appeared among the output genes, together with a splicing factor and an RNA-binding protein.

**Table 4:** **Genes with largest up-regulation over the time course of winter hardening**.

**Gene ID**	**MapMan Bin**	**Description**	**TP1**	**TP2**	**TP3**	**TP4**
AT1G77490	Redox.ascorbate and glutathione.ascorbate	TAPX (THYLAKOIDAL ASCORBATE PEROXIDASE)	1.26	**2.78**	**3.02**	**3.41**
AT1G63940	Redox.ascorbate and glutathione.ascorbate	MDAR6 monodehydroascorbate reductase	1.33	**3.19**	**2.46**	**3.27**
AT4G35090	Redox.dismutases and catalases	CAT2 (CATALASE 2)	**4.83**	**6.98**	**7.34**	**7.91**
AT1G20620	Redox.dismutases and catalases	CAT3 (CATALASE 3)	**5.28**	**10.22**	**10.86**	**11.63**
AT3G02000	Redox.glutaredoxins	ROXY1 disulfide oxidoreductase	**2.54**	**4.67**	**5.27**	**6.22**
AT1G50640	Hormone metabolism.ethylene.signal transduction	ERF3 (ETHYLENE RESPONSIVE ELEMENT BINDING FACTOR 3)	**1.86**	**5.80**	**12.85**	**8.97**
AT5G13200	Hormone metabolism.abscisic acid.induced-regulated-responsive-activated	GRAM domain-containing protein	**3.26**	**8.52**	**14.89**	**12.81**
AT1G53530	Protein.targeting.secretory pathway.unspecified	Protein transport SEC61 gamma subunit	1.90	2.92	8.022	4.88
AT3G48570	Protein.targeting.secretory pathway.unspecified	ARFB1B	**1.68**	**2.60**	**2.66**	**3.08**
AT5G17060	Protein.targeting.secretory pathway.unspecified	AGP16 (ARABINOGALACTAN PROTEIN 16)	1.90	**2.92**	**8.02**	**4.88**
AT2G46330	Cell wall.cell wall proteins.AGPs	SUS4; UDP-glycosyltransferase/sucrose synthase/transferase	**3.29**	**6.467335747**	**7.30**	**5.64**
AT3G43190	Major CHO metabolism.degradation.sucrose.Susy	XTH22 | TCH4 (Touch 4); xyloglucan:xyloglucosyl transferase	**2.07**	**4.03**	**3.73**	**4.26**
AT5G57560	Cell wall.modification	Dormancy/auxin associated	1.35	1.36	**4.23**	**1.88**

Expression values in bold typeface are statistically significant changes.

**Table 5:** **Alpine gene association network details**.

**Gene ID**	**GO: biological process**	**GO: cellular compartment**	**Description**	**Notes**
AT1G13190	No match	No match	RNA-binding (RRM/RBD/RNP motifs) family protein	–
AT1G50640	Negative regulation of ethylene mediated signaling pathway	Nucleus, cytoplasm	Ethylene responsive element binding factor 3	ER to Golgi
AT1G68490	No match	Chloroplast	Unknown protein	–
AT2G01600	Clathrin coat assembly	Golgi apparatus, endoplasmic reticlum	ENTH/ANTH/VHS superfamily protein	Clathrin coat assembly vesicle mediated transport vacuole
AT2G27830	No match	No match	Unknown protein	–
AT2G34310	No match	No match	Unknown protein	–
AT2G43160	No match	Plasma membrane	ENTH/VHS family protein	–
AT3G07950	No match	Endomembrane system	Rhomboid protein-related	Golgi response to salt
AT3G52120	mRNA processing	Nucleus, cytoplasm	SWAP (Suppressor-of-White-APricot)/	Splicing factor
AT4G22330	Ceramide metabolic process	Endoplasmic reticulum membrane	ATCES1; catalytic/Hydrolase, acting on carbon-nitrogen (but not peptide) bonds, in linear amides	–
AT4G32640	Intracellular protein transport	COPII vesicle coat	Sec23/Sec24 protein transport family protein	–
AT4G32760	Intracellular protein transport	Golgi stack, endoplasmic reticulum	ENTH/VHS/GAT family protein	Intra-Golgi vesicle-mediated transport
AT5G11700	No match	Vacuole	Glycine-rich protein	–
AT5G53140	Protein dephosphorylation	Protein Serine/threonine phosphatase complex	Protein phosphatase 2C family protein	–

Table provides further detail about genes depicted in Figure.

## Discussion

With regard to several cell functions, our results show that Sitka spruce needles are surprisingly metabolically active during the fall and early winter, which reflects the need for fundamental remodeling that equips them to survive the stress imposed by freezing temperatures. Numerous cellular processes facilitate this, including the synthesis of cryoprotectant small molecules (di- and oligosaccharides, amino acids, etc.,) and proteins (dehydrins, antifreeze proteins, heat shock proteins). These cryoprotectants are thought to counterbalance the change in osmotic potential between the protoplast and apoplast with extracellular ice formation, to solubilize intra-cellular proteins as the cell dehydrates, and to lower the freezing point of water to prevent intracellular ice formation. However, little is known about the role of cell wall remodeling in this process. As the cell dehydrates and the membrane shrinks correspondingly, it stands to reason that the cell wall must react in a similar way. We previously reported overrepresentation of transcripts in this dataset annotated as involved in cellular transport and trafficking, and sought to better characterize the role that these processes may play in conifer cold acclimation. Although cold acclimation involves substantial metabolic remodeling, which must involve protein and metabolite targeting, little is known about the role of transport in cold acclimation. Studying transcripts in this category in detail led us to conclude that some of this transport activity supports cell wall remodeling, another overlooked area of freezing temperature tolerance. We hypothesized that changes in the cell wall may be required to sustain the severe cellular dehydration accompanying the winter period, and our analysis suggests a coordinated response to do this, from candidate hormone signaling components through synthesis and transport of cell wall substrates.

### Cell walls and secretory pathways

Gene expression patterns observed over the time course of hardening are consistent with cellulose biosynthesis, cell wall modification, increased trafficking of cell wall components to the apoplast, and a concomitant increase in associated signaling processes. It appears that processes associated with the synthesis of cell wall polysaccharides, including cellulose, were quite active over the hardening period. Increases in gene expression were observed for the AtCSL (“CSL”) super family, specifically those encoding proteins that do not catalyze actual cellulose synthesis, but encode glycan synthases that catalyze the synthesis of mannan oligomers (AtCSLA gene family), and members of the AtCSLC family that catalyze production of the glucan backbone of xyloglucans present in primary cell walls (Davis et al., 2010). Proteins encoded by members of these two gene families accumulate in the Golgi lumen, as do many other proteins associated with cell wall synthesis (Parsons et al., 2012). An expression increase was also observed for a member of the AtCSLG gene family (AtCSLG3), which has been reported to respond to low temperatures (Provart et al., 2003). In contrast, members of the AtCSLB and AtCSLE subfamilies were down-regulated over the time course, though no function is known for either of these genes as of yet. In addition, At4g32410 and At5g44030, members of the “true” CES family that responded over the time course of hardening, showed up-regulation. Overall, the evidence points to remodeling of the carbohydrate matrix in the cell wall, with a concomitant up-regulation of cellulose biosynthesis itself.

Expression of genes encoding secretory proteins (such as MUR3) show a similar pattern. Interestingly, the protein encoded by *MUR3* also plays a role in overall endomembrane organization (Tamura et al., 2005), suggesting that this cell wall precursor bestows integrity on the very secretory pathway that apoplastic substrates follow to their destination. In the case of At1g10730, encoding the medium subunit of a clathrin adaptor, the assignment to either the cell membrane or the TGN was made on the basis of its annotation, since clathrin is known to be part of the recruiting process for vesicles derived from those two subcellular locations (Bassham et al., 2008; Parsons et al., 2012). Signaling processes that regulate cell membrane-cell wall transitions may also have been up-regulated. One of the GPI-anchored AGP genes, AGP16, shows an increase over the entire time course of hardening. Since the AGPs are thought to function as signaling molecules (Zhang et al., 2011) by interacting with proteins within membranes that exist in lipid rafts (Zhang et al., 2011), these different members of the AGP gene family may play roles in coordinating the changes in cell composition and function that accompany the hardening process. Direct evidence exists for a role as co-receptor at the cell membrane for AGP18, another GPI-anchored AGP (Seifert and Roberts, 2007). AGP16 itself was found to interact with the protein encoded by At2g26300 (α-subunit of the G-protein), a GTP-binding/GTPase/channel regulator/signal transducer (Klopffleisch et al., 2011).

Taken together, these data suggest that cell wall functional activity continues until TP4, with alterations in the cell wall matrix figuring prominently in these processes. Cell wall components are known to be synthesized and processed in the endomembrane system (Driouich et al., 2012), and it is interesting to note that many of the gene products for which increases in transcript levels were detected are targeted to the endomembrane system, suggesting substantial cold-induced cellular trafficking. Two notable exceptions are AXS1 and AXS2, which are both predicted to be localized to the nucleus and which show increases at TP3 and 4, respectively. It is possible that these genes “moonlight” as regulators of cell wall function or other processes that underlie the acquisition of cold hardiness.

### Role of phytohormone signaling in cell wall remodeling

Ethylene-related genes responded positively over the time course of winter hardening. Three genes annotated as related to ethylene biosynthesis and/or regulation of ethylene levels, ACS12, ACC oxidase, and D-cysteine desulfhydrase (D-CDES), were up-regulated. Interestingly, ethylene is known to participate in the flux control of UDP-D-galactose into cell wall polymers (Seifert et al., 2004), providing a causal link between the apparent increase in ethylene levels and the increases in cell wall-related activities over the time course of winter hardening. ACS12 is now known not to have ACS activity but, rather, to function as an aminotransferase (Yamagami et al., 2003). However, ACC oxidase is enzymatically active and is part of the ethylene biosynthesis pathway. D-CDES has been shown to act as a modulator of ethylene levels (McDonnell et al., 2009). In the case of ERF3, which was up-regulated throughout the time course, and substantially up-regulated by TP4, the encoded protein has been shown to interact with histone deacetylase1 and is thought to contribute to the modulation of stress signal transduction (Song and Galbraith, 2006). ERF12 is inferred by virtue of the presence of the same EAR domain present in ERF3 to act as a transcriptional repressor (Fujimoto et al., 2000; Ohta et al., 2001). ERF1 is an activator of gene expression through binding to GCC boxes (Fujimoto et al., 2000). ERF2 acts as a positive regulator of jasmonic acid (JA) signaling (McGrath et al., 2005), and its down-regulation over the time course of hardening suggests repression of that signaling pathway. There is nothing known about the functions of the universal stress proteins, and PDX 1.2, although a member of the pyridoxine biosynthesis family by virtue of its sequence, does not have enzymatic activity and may act as a regulatory protein (Leuendorf et al., 2010).

Ethylene-related genes that were down-regulated throughout the time course of winter hardening included *HOOKLESS1*, which is thought to act both as a mediator between auxin and ethylene signaling through an unknown mechanism (Chatfield and Raizada, 2008) and to negatively regulate sugar and auxin signaling (Ohto et al., 2006). Two ethylene-responsive members of the MBF gene family, *MBF1B* and *C*, were also down-regulated over the entire time course. To date, the evidence suggests that members of the MBF1 gene family are redundant and acting as repressors of ABA signaling (Arce et al., 2010) through ABR1, an ERF gene. ABR1 does not appear to have responded in the current study. Proteins encoded by the NCED gene family catalyze the key step in the ABA biosynthetic pathway, and numerous studies have demonstrated the induction of NCED genes by abiotic stress. NCED5 is known to be part of the ABA biosynthetic pathway, whereas the function of NCED4 has not been established (Nambara and Marion-Poll, 2005), although it was recently localized to plastoglobuli, within the chloroplast (Lundquist et al., 2012). The down-regulation of NCED5 over the time course of winter hardening makes it likely that ABA levels went down over the time course of the study. Despite this, one gene encoding an ABI5-like protein, a positive regulator of ABA signaling, was up-regulated. In addition, a gene annotated as a possibly ABA-responsive GRAM protein was also massively up-regulated, but there appears to be neither any direct information on its function, nor was it co-expressed with known genes, as displayed in GeneMANIA (data not shown).

This inferred decrease in ABA levels may seem surprising in light of the many reports of the induction of ABA biosynthesis when plants are exposed to cold and drought stress (Iuchi et al., 2001; Tan et al., 2003). However, a similar result was reported in a study comparing adaptive changes to mild drought in young Arabidopsis leaves undergoing cell proliferation and expansion (Skirycz et al., 2010; Verelst et al., 2010) with gene expression responses to the same stress in mature leaves. They found that the “classical” and expected ABA-mediated response occurred in the mature leaves only, whereas ethylene-mediated processes took place in the younger tissue, a result that resonates with the data reported here. In the adaptation study, the authors also observed a comparable up-regulation of genes encoding enzymes that catalyze the biosynthesis of polysaccharide primary cell wall components, in agreement with the data presented in this report. However, those authors reported a down-regulation of genes encoding members of the CES gene family, in contrast to the results reported here. In summary, in the case of ABA, the available data strongly suggest a down-regulation of biosynthesis over the time course of winter hardening, whereas ethylene biosynthesis may have been stimulated along with signaling processes, some related to cell wall modification set in motion by that phytohormone. ABA and ethylene are known to have antagonistic roles in the control of plant cell function (Cheng et al., 2009).

The results from ALPINE provide circumstantial evidence for a regulatory role for *ERF3*, involving vesicular trafficking in the endomembrane system and, by inference, stimulating the synthesis and transport of non-cellulosic cell wall components to their targeted destination across the plasma membrane to the apoplast.

Taken together, the transcriptomic and metabolomic data obtained from Sitka spruce needles over the time course of winter hardening suggest that the transition to a hardened state involves an adaptation process, with trafficking through the endomembrane system, cell wall remodeling, and signaling related to ethylene action, as prominent cellular activities. This adaptation mechanism in Sitka spruce appears to be quite distinct from what is currently understood concerning the responses of angiosperms to severe abiotic stress and may be similar to adaptation mechanisms that occur under more mild stress conditions.

### Conflict of interest statement

The authors declare that the research was conducted in the absence of any commercial or financial relationships that could be construed as a potential conflict of interest.
